# Long Non-Coding RNAs in the Regulation of Gene Expression: Physiology and Disease

**DOI:** 10.3390/ncrna5010017

**Published:** 2019-02-17

**Authors:** Juliane C. R. Fernandes, Stephanie M. Acuña, Juliana I. Aoki, Lucile M. Floeter-Winter, Sandra M. Muxel

**Affiliations:** Laboratório de Fisiologia de Tripanossomatídeos, Instituto de Biociências, Departamento de Fisiologia, Universidade de São Paulo, São Paulo 05508-090, Brazil; juliane.cristina.fernandes@usp.br (J.C.R.F.); stephanie.acuna@usp.br (S.M.A.); juaoki@usp.br (J.I.A.); lucile@usp.br (L.M.F.-W.)

**Keywords:** long non-coding RNA, gene expression, nuclear architecture, chromosome, microRNA, physiology, infectious diseases, biomarkers

## Abstract

The identification of RNAs that are not translated into proteins was an important breakthrough, defining the diversity of molecules involved in eukaryotic regulation of gene expression. These non-coding RNAs can be divided into two main classes according to their length: short non-coding RNAs, such as microRNAs (miRNAs), and long non-coding RNAs (lncRNAs). The lncRNAs in association with other molecules can coordinate several physiological processes and their dysfunction may impact in several pathologies, including cancer and infectious diseases. They can control the flux of genetic information, such as chromosome structure modulation, transcription, splicing, messenger RNA (mRNA) stability, mRNA availability, and post-translational modifications. Long non-coding RNAs present interaction domains for DNA, mRNAs, miRNAs, and proteins, depending on both sequence and secondary structure. The advent of new generation sequencing has provided evidences of putative lncRNAs existence; however, the analysis of transcriptomes for their functional characterization remains a challenge. Here, we review some important aspects of lncRNA biology, focusing on their role as regulatory elements in gene expression modulation during physiological and disease processes, with implications in host and pathogens physiology, and their role in immune response modulation.

## 1. Introduction

The discrepancy of about 20,000 protein-coding genes and over 100,000 different transcripts identified in mammalian transcriptomes highlights the possibility of discovering a novel class of non-translated RNAs [1], beyond those already identified in the 1970s, as part of the translation machinery: ribosomal RNAs (rRNAs) [2] and transfer RNAs (tRNAs) [3]. The advent of next generation sequencing is providing considerable amounts of new information about genomic organization and gene expression regulation [4,5]. These data have been helping to revise our understanding of the current genome and gene annotations [5]. The transcriptome data from cellular lineages and human tissue samples showed that at least 60% of the genome is expressed as primary or processed transcripts, much more than previously predicted [6]. This analysis revealed that thousands of unannotated RNAs may act as non-coding regulatory elements in gene expression or originate as small RNAs [6].

**Figure 1:**
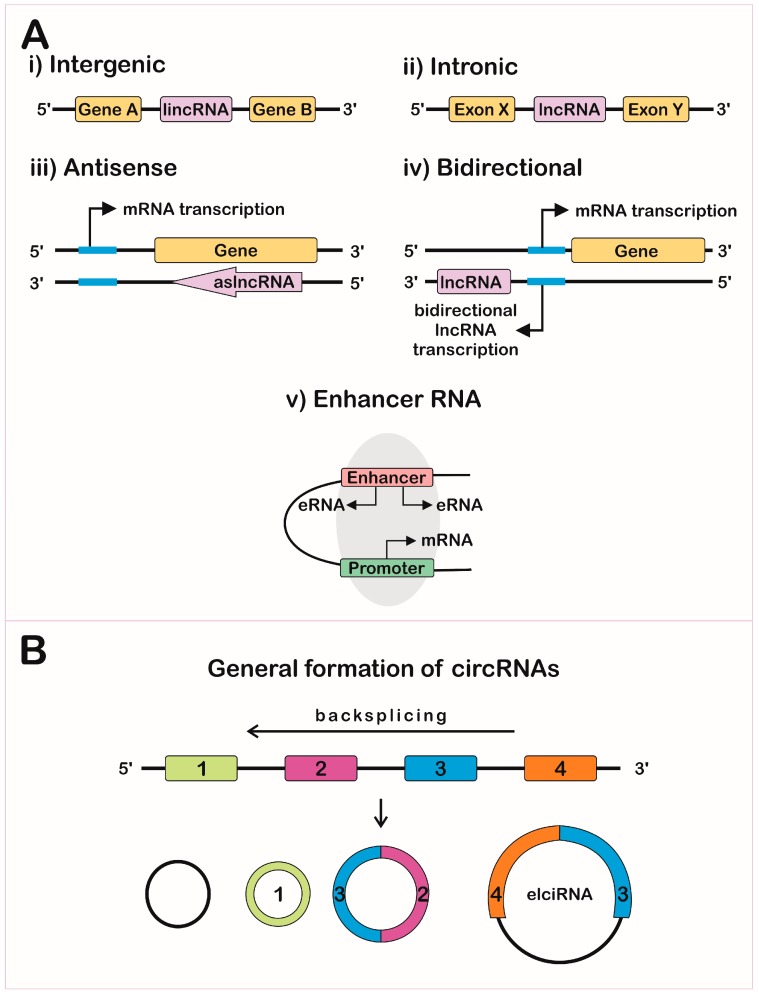
Schematic representation of the genomic loci of different long non-coding RNA (lncRNA) and circular RNA (circRNA) biogenesis. (**A**) lncRNA classification depends on the genomic position: (i) intergenic RNAs (lincRNAs) are located between two protein coding-genes, (ii) intronic lncRNAs are positioned within an intronic region of a protein coding-gene, (iii) antisense lncRNAs (aslncRNAs) are transcribed from complementary strands, (iv) bidirectional lncRNAs originate from bidirectional transcription of protein-coding genes, and (v) enhancer RNAs (eRNAs) originate from enhancer regions and mediate transcription factor positioning into protein coding-genes promoters. (**B**) circRNAs are lncRNAs that undergo back splicing and can originate from transcripts containing only intronic, one or more exonic, or both intronic and exonic fragments (elciRNAs).

Non-coding RNAs (ncRNAs) can be grouped according to their length, localization, and/or function: long non-coding RNAs (lncRNAs), microRNAs (miRNAs), small interfering RNAs (siRNAs), small nucleolar RNAs (snoRNAs), small nuclear RNAs (snRNAs), and PIWI-interacting RNAs (piRNAs) [7,8]. The lncRNAs are larger than 200 nucleotides and can be subdivided according to their biogenesis loci: intergenic lncRNAs (lincRNAs) [9], intronic lncRNAs, antisense lncRNAs (aslncRNA or natural antisense transcripts, NATs) [10], bidirectional lncRNAs, and enhancer RNAs (eRNAs) (Figure 1A). 

The lncRNA biogenesis is mostly similar to messenger RNA (mRNA), since this process is also mediated through RNA polymerase II. In addition, lncRNAs can appear with or without polyadenylation [11,12], alternative cleavage, alternative polyadenylation, and alternative splicing [13], leading to different isoforms from the same locus [11,14]. 

The lncRNAs genes share features with coding-genes promoters and can be regulated by some transcription factors (TFs), such as p53, nuclear factor-kappa B (NF-κB), Sox2, and Oct4 [15]. Some lncRNAs present unique requirements for their proper expression and functionality, which can be mediated by cMyc TF and Dicer, a key enzyme involved in miRNA processing [16]. The antisense lncRNAs originate from the complementary strand of protein-coding genes [10,17]; about 30% of human annotated genes have an antisense component that highly impacts gene profile expression [18]. Divergent transcription can occur when the RNA polymerase II is recruited in the antisense strand at an upstream site of the protein coding gene promoter, but only a fraction of them generate functional transcripts, called bidirectional lncRNAs [17,19,20]. Evaluation of lncRNAs in human and murine embryonic stem cells showed that over 60% were divergently transcribed and the changes are coordinated with cognate protein coding genes during differentiation [21]. Cap analysis of gene expression (CAGE) showed that eRNAs are bidirectionally transcribed and capped [22], but non-polyadenylated and non-spliced, depending on the integrator complex for the 3’ end cleavage of the transcript [23]. 

Circular RNAs (circRNAs) were predicted to be the most abundant isoform compared to the linear transcripts [24]. The circRNAs can be formed via regular splicing (intronic circRNAs) [25] and via non-canonical splicing, joining the splice donor at an upstream acceptor site (backsplicing) (Figure 1B) [24]. Some isoforms of circRNAs can be derived from circularization of an intronic fragment with their neighbor exons, forming the exon-intron-circRNA (elciRNAs), which can associate with RNA polymerase II and with U1 snRNA, increasing the transcription of their parental genes in cis [26].

The highly intricate mechanisms to regulates lncRNA degradation leads to specific expression in different cellular types and seems to be subjected to the same mechanisms correlated with mRNA decapping [27], or alternatively through nonsense-mediated decay (NMD) [28], a process that depends on ribosome recruiting and that will be described further in this review for the translational potential of lncRNAs. 

Expression of lncRNAs can be detected from uni- to pluri-cellular eukaryotic organisms, although the processing and mechanisms of action can differ [29]. The most recent NONCODE database source points to over 100,000 lncRNAs in the human genome, but this number seems to be underestimated [1,30,31].

Cellular and temporal specificity drives the mechanism of action of lncRNAs and their simultaneous impact in diverse target genes [32,33,34]. Long non-coding RNA can regulates neighbor protein-coding genes expression and thus contribute to the mRNA and protein content in the cell [32,35]. For instance, in the analysis of T lymphocytes transcriptomes, 1500 lncRNAs were identified, showing that 50% appeared to be exclusively expressed in this cell population, whereas only 7% of the mRNAs are stage- or lineage-specific, suggesting lncRNAs as strong components in cell identity [36].

Similar to protein domains, lncRNAs can interact with nucleic acids or proteins through base-pairing or structural recognition, respectively, so a single lncRNA molecule is able to interact with diverse macromolecules [8]. This interaction is mediated by ribonucleoprotein complexes in which lncRNAs are associated with proteins [8]. A 100-ribonucleotide hairpin chain can interact simultaneously with more proteins than peptide domains of 100 amino acids interacting with other proteins. This can be indicative of possible evolutive advantages in regulation mediated by RNAs [37].

**Figure 2:**
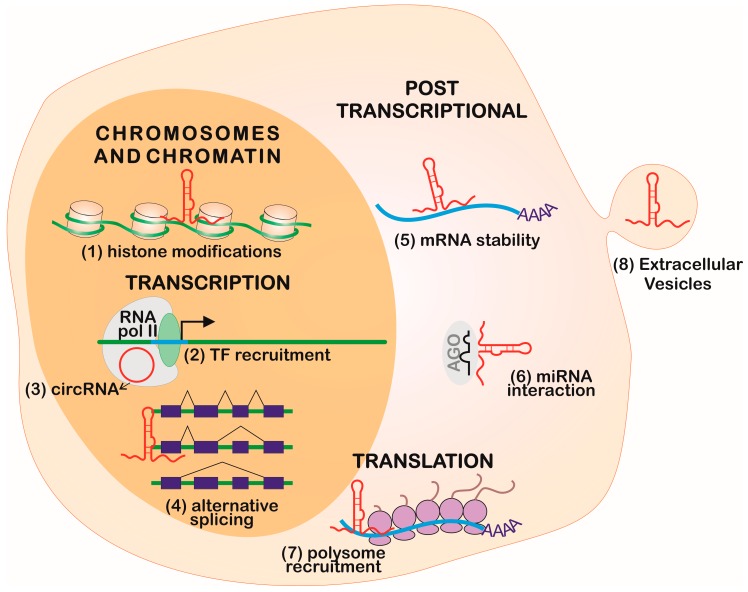
LncRNA participation in gene expression regulation. Many types of lncRNA controls can occur to regulate gene expression, such as: (1) chromatin and chromosome condensation through histone modifications, (2) transcription factors (TF) direct recruitment, (3) binding to RNA polymerase (pol) II, (4) alternative splicing, (5) mRNA stability, (6) miRNA availability, (7) polysomes recruitment, and (8) modulation of gene expression in neighbor cells through packaging into extracellular vesicles.

Long non-coding RNAs are found within the nucleus, nucleolus, cytoplasm, and even in the mitochondria [38,39], and its localization is a good indicator of their mode of action [1], as shown in Figure 2. The RNA-Seq obtained from these compartments showed a strong enrichment of lncRNAs in the nucleus, specifically associated with chromatin in several cell lines when compared to mRNAs [5]. Recent studies estimated that more than half of the expressed lncRNAs are in the cytoplasm, associated with polysome fractions, controlling stability, and translation of mRNAs [40]. Altogether, these data indicate that lncRNA localization depends on the motifs signatures: protein signal-peptides, nuclear-restricted lincRNA BMP/OP-responsive gene (BORG) [41], and Alu-related sequences in a more generally-spread nuclear retention mechanism [42].

Apart from lncRNA localization, secondary and tertiary structures of lncRNA are also important for their mechanism of action. In 2010, a group used a high-throughput approach describing the whole transcriptome structure of *Saccharomyces cerevisiae* at nucleotide resolution [43]. These conformations have been unveiled using diverse techniques, such as fragmentation sequencing (FragSeq), which is based on sequencing of fragments digested by single- or double-strand specific nucleases [44], which can be useful in the description of RNA molecular structure and in the identification of folding domains that mediate interaction with other macromolecules. This is also important in the understanding of lncRNA evolution once there is a low level of primary sequence conservation [45,46], but also conservation at the stem-loop structure level, maintaining the functionality of these molecules [47].

The lncRNA structural changes can also regulate the availability of recognition sites for RNA binding proteins through thermodynamic adjustments in the hairpin stability [48,49]. The most common RNA chemical modifications are the exchange of adenosine to inosine, catalyzed by adenosine deaminases, and the reversible modifications by N6-methyl-adenosine (m6A) methylation [50]. Besides regulating function, these modifications are essential for the recognition of the RNAs as endogenous and non-pathogenic molecules, whereas non-modified RNAs are capable of stimulating the immune response mediated by toll-like receptors (TLRs) [31]. Some tools can predict sites responsible for editing and the impact on structure and function, such as interaction with miRNAs [51].

## 2. Gene Expression Regulation Levels

The regulation of gene expression in eukaryotes is complex and compartmentalized [52]. It can occur in multiple steps, such as in the chromatin organization, transcription machinery recruitment, mRNA processing and its delivery to the cytoplasm, mRNA half-life, translation, and posttranslational processes, which can be interfered with by lncRNAs [31,53], as represented in Figure 2. These molecules can also be secreted within extracellular vesicles, modulating the gene expression in its environment [54].

### 2.1. Chromosome and Chromatin Structure

The idea that RNA can be a chromatin-associated structural component was corroborated by the description that, there, the amount of RNA is twice as high as the DNA associated with the chromatin structure [55]. Many studies identified several types of RNAs related to this function, such as snRNAs, and lncRNAs, such as the X inactivation-specific transcription (XIST), AIR, and H19, were associated with heterochromatin formation and imprinting [8]. Additionally, lncRNAs that are expressed only in embryonic stem cells interact directly with the chromatin, then modulate gene expression and the maintenance of pluripotency [56]. The lncRNA interaction with DNA can occur by sequence complementarity to a single-stranded fragment of DNA or allocation in the helix [31]. Additionally, eRNAs can execute their function by mediating chromosomal looping together with the mediator complex [57]. As such, lncRNAs are related to a general structuration of the genome, organizing nuclear architecture, and consequently, gene expression [58], as shown in Figure 2.

### 2.2. Transcription

At the transcriptional level, the promoter region of a lncRNA sequence, regardless of its synthesis, can act as an enhancer, characterizing a *cis* regulation [59]. The NAT asOct4-pg5 can indirectly regulate epigenetic markers through the RNA/DNA binding protein PURA (purine-rich element binding factor A), which reduces transcription from the protein-coding sense transcripts and simultaneously represses other NATs in a negative-feedback loop [60]. Some ncRNAs can interact directly with the transcription machinery, as shown by circRNAs that directly interact with RNA pol II, according to crosslink followed by immunoprecipitation assays (Figure 2) [31]. Additionally, eRNAs can bind to transcription factors, positioning them in specific promoters [61]. Other lncRNAs can regulate transcription, controlling DNA methyltransferases recruitment, TFs, zinc-finger proteins, and others transcription regulators [1]. 

### 2.3. Post-Transcriptional Regulation

#### 2.3.1. Long Non-Coding RNA and MicroRNA Interplay

**Figure 3:**
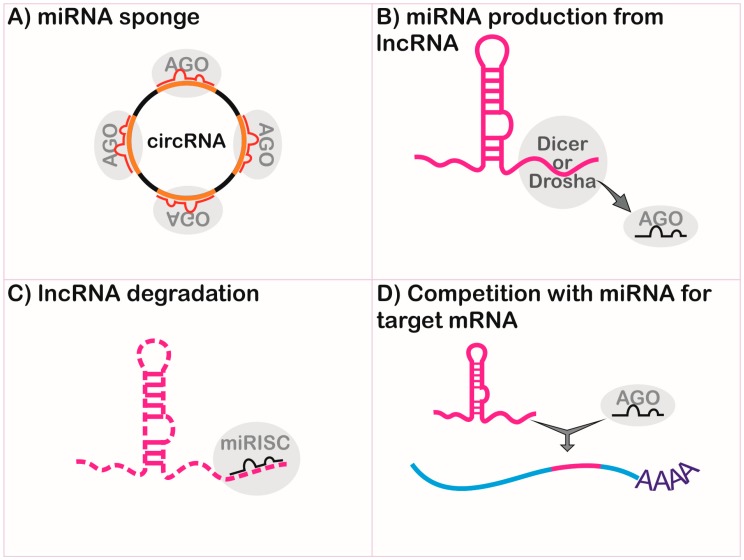
Interaction between microRNAs (miRNAs) and lncRNAs. (**A**) lncRNAs can act as miRNA sponges in both linear or circular forms, thus reducing interaction between miRNA-target mRNA; (**B**) lncRNA can harbor miRNA precursors, producing mature miRNAs after Dicer and/or Drosha cleavage; (**C**) miRNA can target lncRNA for degradation, similar to 3’ untranslated region binding to mRNAs; and (**D**) lncRNA–miRNA competition for the mRNA binding site. circRNA—circular lncRNA; AGO—argonaute proteins; miRISC—miRNA-induced silencing complex; Dicer—endoribonuclease; Drosha—endoribonuclease.

Different classes of ncRNAs can interact through sequence complementarity by executing coordinated functions. The most remarkable interplay occurs between lncRNAs and miRNAs in the regulation of gene expression (Figure 2 and Figure 3).

Long non-coding RNAs can be endogenous competitors RNAs (ceRNAs), also called miRNA sponges, by presenting binding sequences for miRNAs [62,63], and can impair the functional interaction of miRNA and mRNA by interference in the gene regulation, as shown in Figure 3A. Both linear and circular isoforms can exert this function [62]. The first description of competition between these molecules involved the naturally-expressed circular RNA sponge for miR-7 (ciRS-7), via miRNA-dependent binding to argonaute (AGO) proteins [62]. During myogenesis, the linear transcript named lnc-MG competes with miR-125b, controlling insulin-like growth factor 2 (IGF-2) levels [64]. In hepatitis C virus infection, lncRNA-ATB is activated by transforming growth factor-beta (TGF-β) and competes with miR-425-5p, upregulating the TGF-β type II receptor (TGF-βRII) and SMAD family member 2 (SMAD2), promoting liver fibrosis [65].

Long non-coding RNAs can be precursors of miRNAs (Figure 3B) and can regulate different points of miRNA biogenesis, acting on microprocessor activity to finish the primary transcript in a mechanism independent of polyadenylation [66]. For example, the lncRNA named LOC554202 originates miR-31 and is important in preventing metastasis in breast cancer [67]. The lncRNA deleted in lymphocytic leukemia 2 (DLEU2) harbors miR-15a/16.1 within its third intron and expression dysfunction is associated with lymphocytic leukemia [68,69]. The lncRNA colon cancer-associated transcript 2 (CCAT2) blocks the maturation of miR-145 by inhibition of Dicer cleavage and cytoplasm export [70]. The miR-221 and miR-222 are co-transcribed with lnc-Ang362, an angiotensin II-upregulated lncRNA in endothelial cells [71]. 

MicroRNAs can regulate the stability and half-life of lncRNA (Figure 3C) [72]. The miR-152 is a negative regulator of the XIST lncRNA in a repression feedback loop in glioblastoma cells [73]. Both miR-101 and miR-217 negatively regulate metastasis associated with lung adenocarcinoma transcript 1 (MALAT1) in carcinoma cells [74], and miR-449a inhibits the expression of nuclear enriched abundant transcript 1 (NEAT1) lncRNA in lung cancer [75].

Long non-coding RNAs can compete with miRNAs for the target site of mRNA (Figure 3D). This is demonstrated by antisense transcript for the β-site amyloid precursor protein cleaving enzyme 1 (BACE1-AS), which binds to its sense partner in the miR-485-5p recognition site, thus impairing its function, which is implicated in Alzheimer’s disease [76]. 

Some lncRNAs can exert multiple functions, as described for lncRNA H19, which acts as a molecular sponge for let-7 [77] and is a precursor of miR-675 [78], mediating muscular differentiation and regeneration [77,78]. The lnc-MD1 sequestrates both miR-133 and miR-135, allowing mastermind-like-1 and myocyte-specific enhancer factor 2C expression during myogenesis [79]. miRNA clusters can comprise functional lncRNA, such as the increased levels observed for the miR-99a, let7c, and miR-125b-2 cluster in leukemic cells lines, which comprise a lncRNA gene MIR99AHG in the intronic region of the *hsa21* gene and the miR-125b-1, let-7a-2, and miR-100 cluster, forming MIR100HG lncRNA within the hsa11 locus [80].

#### 2.3.2. Alternative Splicing

More than half of intron-containing genes in animals and plants can be alternatively spliced, generating different mature mRNA isoforms [81]. Diverse ncRNAs are involved in this process, such as the well-described uridine-rich small nuclear RNAs (U snRNAs) that act as a ribonucleoprotein complex in all splicing processes [82]. Recently, the lncRNAs NEAT1 and MALAT1 were observed colocalizing with nuclear speckles containing the splicing factor SC35 [83]. Also, MALAT1 regulates phosphorylation and activation of serine/arginine (SR) splicing factors [84]. From these observations, lncRNAs are described as interacting with splicing factors, composing duplexes of pre-mRNAs and antisense lncRNAs or chromatin remodeling, which can directly influence the transcriptional rate by RNA polymerase II and modulate splicing [85,86]. An example of this mechanism is described in different human cell lines in which the first apoptosis signal (Fas)-antisense lncRNA-SAF interacts with the human splicing factor SPF45, leading to exclusion of exon 6 during splicing of Fas and production of a soluble Fas protein, thus preventing Fas-FasL-mediated apoptosis [87]. 

#### 2.3.3. Messenger RNA Stability

Long non-codin RNAs have properties capable of mediating the nonsense-mediated mRNA decay (NMD) pathway, in a miRNA-independent way. This function is exemplified by the complex formed by lncRNA half-STAU1-binding site RNAs (*1*/*2-sbsRNAs*) with the target mRNA, creating a double-stranded (ds) transactivation motif that interacts with the STAU1-dsRNA binding protein, leading to mRNA degradation [88]. Some NATs can also decrease the stability of the protein-coding sense transcript [89,90]. Alternatively, some aslncRNAs can increase pair stability, as observed for β-secretase-1 in Alzheimer’s disease [91]. A study demonstrated a tumor suppression role for the PDCD4 antisense transcript, as it stabilizes its cognate mRNA by forming a duplex structure that regulates the association with mRNA decay factors [92]. 

### 2.4. Translation 

Translation processes can be facilitated or repressed by lncRNAs, as shown by the dopaminergic neuron-specific expression of *Uchl1* (ubiquitin carboxy-terminal hydrolase L1), which is regulated by its antisense transcript (AS *Uchl1*), which recruits polysomes by the repetitive domain SINEB2 to promote a cap-independent translation [93]. lncRNA-p21 was demonstrated to repress translation of JunB and β-catenin mRNAs by recruiting translational repressors [94,95].

## 3. Physiological Conditions and Disease

The roles of lncRNAs in genome integrity and gene expression have demonstrated the relevance of these molecules for physiological and pathological conditions [96,97,98]. Here, we review the involvement of lncRNAs in chromosomal compensation, imprinting, chronic diseases, immune response process, and in some pathogens. 

### 3.1. X Chromosome Dosage Compensation

A classic example of chromatin structure regulation mediated by lncRNA, described in the 1990s, is the dosage compensation of the X chromosome in females during embryogenesis, since one of them is inactivated equalizing the dosage as in XY males [99,100]. XIST is expressed exclusively from the inactivated X chromosome (Xi), producing a lncRNA that binds directly to polycomb repressive complexes 1 and 2 (PRC1 and PRC2, respectively) [76]. This mechanism leads to the formation of heterochromatin with the recruitment of histone methylases of the H3K27me3 type [101]. Additionally, a repressive nuclear environment is formed by the depletion of TFs, RNA polymerase II, splicing factors, and nascent RNAs, mainly in the pericentromeric region [102]. The specificity and recognition motifs of XIST to the Xi chromosome have been extensively studied, but it was not possible to determine consensus domains, suggesting that these lncRNAs can act through multiple interactions [103]. Colocalization studies demonstrated ligation mainly in repetitive regions of the X chromosome, which are not coding genes, and long interspaced elements (LINEs), abundant in the X chromosome [104]. Ectopic expression of XIST in autosomal chromosomes can lead to heterochromatin formation [105]. The female heterochromatinization of the X chromosome is complex and depends on nuclear architecture, epigenetic modifications, and non-coding RNAs [106].

In *Drosophila melanogaster*, the dosage compensation is performed via male X chromosome hyperactivation through histone acetylation [107,108,109]. This mechanism is also mediated by lncRNAs, the roX1 and roX2 that play redundant functions and act together with a complex of five proteins exclusive to males, and are essential for survival [110,111].

### 3.2. Imprinting

In mammals, epigenetic markers can regulate maternal or paternal chromosomes expression; this phenomenon of exclusive or differential expression is called imprinting and can be mediated by lncRNAs. The genes *Air* (antisense Igf2r RNA) and *Kcnq1ot1* (lincRNA from *Kcnq1* gene) are transcribed from the paternal chromosome and recruit G9a methylase for histone modification silencing exclusively in the maternal genes [112]. The regulation can occur in *cis* via Air binding to the promoter region of the paternal *Igf2r* gene, silencing its expression by inhibiting the recruitment of RNA pol II [113]. Besides the potential for chromatin silencing by lncRNAs, these molecules can act as enhancers, for example, the lncRNA HOTTIP (HOXA distal transcript antisense RNA) interacts directly with components of the histone activation machinery through H3K4me3 modifications [94].

### 3.3. Chronic Diseases

Besides all physiological mechanisms described, the dysregulation of the expression of lncRNAs can occur during chronic multifactorial diseases [114]. Most studies describe the function of lncRNA in cancer, correlating it with diverse TFs and molecules that regulate the cell cycle, as well as linking it with the processes of pluripotency and differentiation [56]. A larger number of human diseases involve lncRNAs and more than 900 lncRNAs, having an important role in complex diseases, such as cancer and cardiovascular and neurological diseases, according to the experimentally supported data in the lncRNA disease database [115].

Long non-coding RNAs are correlated with different aspects of complex diseases and differential expression was observed in patients with neurodegeneration, such as Alzheimer’s [116], Huntington’s [117], and Parkinson’s [118] diseases; schizophrenia [119,120]; and autism spectrum disorders [121,122]. Dysregulation is frequently described in cardiovascular diseases, such as during chronic heart failure [123], diabetic cardiomyopathy [124], atherosclerosis [125], and infarction [126]. The complexity of lncRNA regulation and specificity are being revealed as important determinants in diabetes mellitus [127].

**Figure 4:**
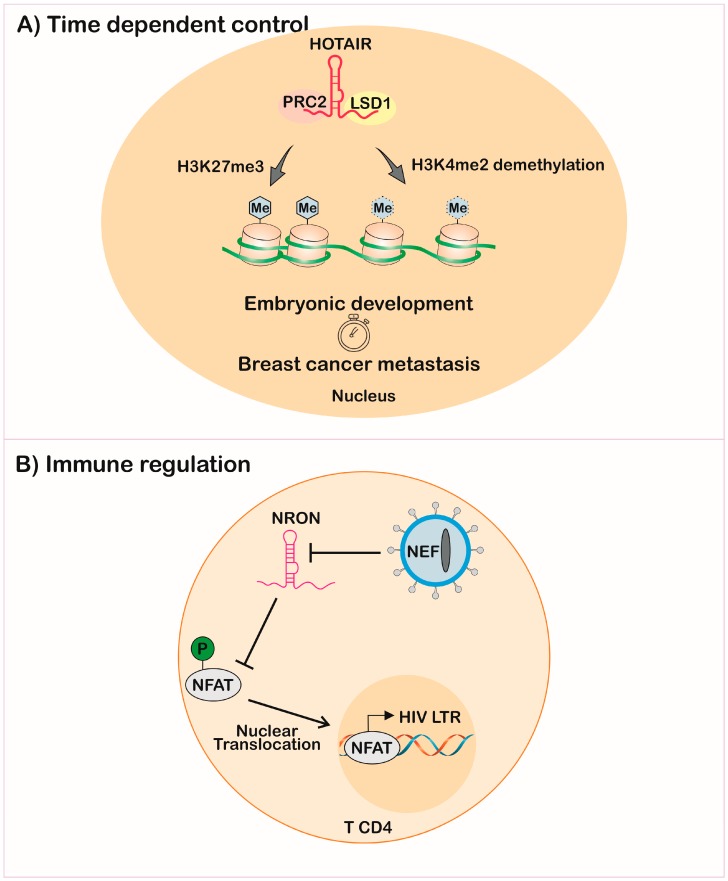
Patho-physiological implications of lncRNAs expression in a time- and infection-dependent manner. (**A**) Expression of HOTAIR complexed with PRC2 and LSD1 leads to trimethylation of the histone 3 at the lysine 27 and demethylation of the lysine 4, controlling gene expression during embryonic development, but leading to breast cancer metastasis when expressed in adult breast tissue; (**B**) disruption of the NRON lncRNA expression in T CD4+ lymphocyte by the HIV Nef protein leads to NFAT translocation to the nucleus and LTR transcription. Abbreviations: HOTAIR—*Hox* transcript antisense intergenic RNA; PRC2—polycomb repressive complex; LSD1—lysine-specific histone demethylase 1; Me—methyl groups; H—histone; K—lysine; NEF—negative regulatory factor, NRON—non-coding repressor of NFAT; NFAT—nuclear factor of activated T-cells; HIV—human immunodeficiency virus; LTR—long terminal repeat; T CD4—CD4 T lymphocytes.

The same innate mechanisms of action of lncRNAs can lead to malignant transformation of the cell if expressed in moments distinct from the physiological ones. In mammals, the lncRNA HOTAIR (*Hox* transcript antisense intergenic RNA) has two chromatin modifier recruitment domains, ensuring H3K27 methylation by PRC2 and H3K4 demethylation by lysine-specific demethylase 1 (LSD1). This mechanism is important during embryonic development in regulating homeotic genes together with other HoxC cluster components [128,129]. However, the dysregulation of HOTAIR promotes transformation and metastasis in diverse cancer models [122,130] (Figure 4A). 

The dysregulation of Foxp3 long intergenic non-coding RNA (FLICR) can decrease FoxP3 levels and; therefore, T regulatory cells (Treg) control, leading to autoimmunity [131], showing that lncRNA controls both pro- and anti-inflammatory processes, as next described for infectious diseases. Other autoimmune diseases may also involve lncRNAs, such as psoriasis [132], rheumatoid arthritis [133], and Chron’s disease. [134] 

### 3.4. Immune Response Against Infectious Diseases

Complex relationships between host and pathogens often use gene expression control of the host cell in the evasion of the immune response. lncRNAs have been related to the fine-tuned regulation of inflammatory processes [135,136]. The nuclear architecture was found to be an essential component of trained immunity transcription of several genes mediated by lncRNA-driven chromatin labels, named immune gene-priming lncRNA (IPLs) [137]. This occurs in responses mediated, for example, by tumor-necrosis factor (TNF), which explain the simultaneous upregulation of several cytokines and chemokines [137]. The mechanism relies on trimethylation of histone 3 at lysine 4 (H3K4me3) on the promoters of trained immune genes, in which the modification is mediated by topologically-associated domain (TAD)-transcribed lncRNAs recruiting mixed lineage leukaemia (MLL) methyltransferases and other transcriptional regulators, leading to RNA polymerase II recruitment and activation [137].

The activation of TLR4 by lipopolysaccharide (LPS) recognition led to differential expression of 221 of 989 evaluated lncRNAs in monocytes [138]. Diverse lncRNAs are capable of interfering in NF-κB signaling, one of the main TFs mediating the inflammatory response. Ma et al. demonstrated that lncRNA-Tnfaip3 acts as a coregulator of NF-κB in murine macrophages [139]. lncRNA Lethe is induced through TNF-α signaling and inhibits transcription of NF-κB-dependent genes by association with *RelA*, such as *Il6*, *Il8,* and *Nfkbia* [140]. The lncRNA THRIL (TNF-α and hnRNPL related immunoregulatory lncRNA) is induced by TLR2 and regulates TNF-α expression [141]. Sequestration of the p50 subunit by the lncRNA PACER enables the formation of the activation dimer and increases Cox2 transcription [142]. Stimulation with Pam_3_CSK_4_ (TLR2), LPS (TLR4), and R848 (TLR7/8) mediates MyD88 and NF-κB transcription, inducing a co-expression of Cox2 and lncRNA-Cox2 [143]. Activation of the TLR-pathway also modulates miRNA [144]. The NAT AS-IL1α regulates IL-1α transcription and is expressed at low levels in resting macrophages and is induced by *Listeria monocytogenes* infection or TLR ligands followed by NF-κB activation [145]. Other regulated pathways by lncRNAs include the Janus kinase/signal transducers and activators of transcription (JAK-STAT) and mitogen-activated protein kinase (MAPK) pathways [146,147].

Similar to miRNA studies, an increasing number of dysregulation processes during infections have been showing molecular markers of lncRNAs associated with diverse pathogens [148,149,150]. lncRNAs are modulated during infection with *Mycobacterium tuberculosis* [115,151,152,153,154,155], *Salmonella typhimurium* [156,157], *Escherichia coli* [158], *Helicobacter pylori* [159,160], and *Campylobacter oncisus* [161].

The measurement of lncRNAs after infection of T-cell lines with the human immunodeficiency virus (HIV) showed regulation of host-lncRNA. A previous study also linked HIV infection with upregulation of lncRNA NEAT1 [162]. The regulation of the non-coding repressor of NFAT (nuclear factor of activated T-cells) named NRON was decreased by the HIV Nef-early replication protein and increased by HIV Vpu-late replication, showing that viruses’ proteins can modulate lncRNA expression on host cells. NRON was shown to be an immune-response subversion molecule, once it impairs expression of genes with NFAT-dependent promoters, through impairment of its nuclear translocation [163]. This model showed that HIV downregulated lncRNA, promoting the NFAT translocation and, consequently, its replication through the long terminal repeat (LTR) expression, evidenced by a higher number of virions in the supernatant of NRON knockdown cells [164] (Figure 4B).

The first report on modulation of lncRNA expression during a protozoan parasite infection was recently reported. Human foreskin fibroblasts infected with *Toxoplasma gondii* presented 996 lncRNAs differentially expressed in comparison with non-infected cells [165]. Additionally, co-expression networks revealed that these molecules are correlated with modulated mRNAs involved in immune response, mainly proinflammatory cytokines [165]. High or low virulent strains of *T. gondii* also modulated lncRNAs during infection of mouse bone marrow-derived macrophages [166], corroborating the immune response modulation through lncRNA profile regulation. 

Little is known about the mechanism of these lncRNAs during infections, and many models, mainly parasitic and protozoan infections, have not yet been investigated, providing an interesting field of research.

## 4. Long Non-Coding RNA Expression in Pathogens 

Some studies have provided new insights into pathogen genome and transcriptome data. These data describe pathogens with tightly-tuned regulation, whereas the regulatory elements themselves are still largely uncharacterized. 

Thousands of lncRNAs were identified within the *Schistosoma mansoni* [167] and *Schistosoma japonicum* [168] genomes. Expression analysis revealed specificity along different stages of the parasite, indicating an important modulatory function of lncRNA in the lifecycle of a parasite [167], as well as sex-specific- or drug-resistant-related lncRNAs [169]. 

The first study in pathogenic fungus was on *Cryptococcus neoformans* lncRNA RZE1, which participates in the morphological transition from yeast to hypha, essential for pathogenesis [170].

On the side of the protozoans, NATs were described for *Plasmodium falciparum* [171,172], *Trypanosoma brucei* [173], *Leishmania major* [174], *Leishmania infantum* [175], *Giardia lamblia* [176,177], and *Trichomonas vaginalis* [178], although no consensus exists on labelling these transcripts as lncRNAs, as many studies retain the ncRNA definition.

Several lncRNAs were identified in the malaria parasite *P. falciparum,* which differs in expression during different stages of the life cycle [179,180], with some associated with the telomere-associated repetitive elements (TARE) region [181]. These molecules were associated with the regulation of expression of virulence-related genes [182], especially with the complex regulation of the monoallelic expression of the *var* genes family [183].

The first evidence of ncRNAs in *Leishmania* showed RNA polymerase II-dependent transcription from subtelomeric tandem repeats, 3´-end processing by polyadenylation, expression of sense and antisense transcripts in a tightly regulated manner, cytosolic localization, and potential association with a small RNP complex [175,184]. The identification of ncRNAs through computational analysis was described in *L. major* and *L. donovani* localized in untranslated regions (UTRs), suggesting UTR transcripts as a common feature in *Leishmania* [185]. Around 11,243 ncRNAs of different classes were identified along the *L. braziliensis* genome by algorithm prediction and confirmed by RNA-seq analysis, suggesting that they are real transcripts [186]. *Leishmania amazonensis* promastigotes express a ncRNA among the top five transcripts differentially expressed in the comparison of wild type parasites with parasite arginase knockout, an enzyme essential to parasite survival and differentiation [148,187,188], indicating that this ncRNA may has a role in the gene expression modulation in different metabolic contexts, such as arginase activity [189].

The transcriptome of *T. brucei* revealed ncRNAs without open reading frames (ORFs) or having the potential to codify small peptides [190], but no study has demonstrated functionality for such transcripts.

lncRNA description in eukaryotic pathogens is poorly annotated, probably due to insufficient genome investigation and annotations, mostly because of complex genomic arrangements. However, the characterization of these molecules could improve the knowledge of fungi and parasite biology, genomic organization, and transcriptional expression and regulation, possibly providing potential drug targets.

The lncRNA function in virus replication has been extensively studied, as reviewed by Chavez-Calvillo [191], showing that lncRNA encoded by Gammaherpesvirus, a subfamily of Herpesviridae, is directly involved in the scope of the immune response and viral lytic reactivation, promoting virus genes expression and DNA amplification in the assembly and release of progeny virions [192], as exemplified by Epstein–Barr virus (EBV)-encoded RNA1 (EBER1) [193], EBV-encoded RNA1 and 2 (EBER1 and EBER2, respectively) [194], and Kaposi’s sarcoma-associated herpesvirus (KSHV) polyadenylated nuclear (PAN) RNA [195]. 

## 5. Novel Perspectives

lncRNAs comprise a field of study that has gained visibility and has become attractive to the scientific community. Findings have evidenced the complexity of mechanisms of action of these molecules and that their functions surpass those initially described. Many complicating factors have been added to the study of these molecules, including the protein coding potential of some molecules annotated as non-coding. This has attracted those interested in gene expression regulation. The clinical importance of these findings is being exploited to find novel biomarkers and drug targets due to both the specificity of lncRNA expression and the potential for plasma localization when secreted in extracellular vesicles (EVs).

### 5.1. Long Non-Coding RNAs as Biomarkers

The importance of understanding lncRNA mechanisms extends beyond the description of gene regulation and can be used as diagnostic markers or drug targets as well as prognostic markers. One potential diagnosis marker is the prostate cancer-associated transcript 1 (PCAT-1), which can be identified in the urine of prostate cancer patients [196]. Still on prognosis, the lncRNAs named CAT104, LINC01234, and STXBP5-AS1 have clinical significance in predicting survival of breast cancer [197]. lncRNAs have been proposed as therapeutic targets, and difficulties reside within complete understanding of their mechanism of action [198,199]. 

Many cancer models were demonstrated to release EVs containing different sets of ncRNAs, such as miRNAs and lncRNAs (Figure 2). Some specific transcripts are abundant in EVs, indicating that they may play a role in neighboring cells. This was shown in a colorectal cancer model where miRNA [200], lncRNA [201], and circRNA [202] were selectively exported to EVs and were shown to be not simply correlated with cytosolic RNA pool levels. During hypoxia, hepatocellular cancer cells contained the linc-RoR (regulator of reprogramming), thus decreasing miR-145 and hypoxia-inducible factor 1 alpha (HIF-1α) in recipient cells [203]. This feature demonstrates the potential for lncRNAs as biomarkers, as shown in liver cancer [204].

### 5.2. Micropeptides

The postulate that lncRNAs do not codify proteins was questioned by the description of functional peptides codified by small ORFs. Once the presence of initiation codons followed by stop codons of translation is frequent in a genome, it was necessary to apply appropriate techniques to the identification of peptides codified by small ORFs [205]. Some studies demonstrated the translation and functionality of peptides from the sequencing of RNA fragments bound to ribosomes, followed by the identification of micropeptides through mass spectrometry together with an evolutive analysis, demonstrating the conservation of these sequences [206,207]. This discovery evidenced the importance of verifying the translation potential in these molecules. The products are called micropeptides, small peptides (sPEPs), or peptides of small ORFs (smORFs), and their role in physiological and pathological processes demonstrated their biological importance.

Some studies detailed mechanisms of regulation of muscular function, characterized by contraction and relaxing mechanisms, by micropeptides codified by lncRNAs. Myoregulin (MLN) interacts with the sarcoendoplasmic reticulum calcium transport ATPase SERCA, preventing calcium uptake (Ca^2+^) to the sarcoplasmic reticulum in skeletal muscle and; therefore, knockout mice with this gene have a higher potential of muscular function [208]. The micropeptide DWORF increases SERCA activity by withdrawing the inhibitors phospholamban, sarcolipin, and myoregulin. Knockout mice manipulated by CRISPR/Cas9 showed a reduction in SERCA activity and retarded relaxation of muscle fibers [209]. The lncRNA LINC00961 produces a polypeptide named SPAR (small regulatory polypeptide of amino acid response) that inhibits mTORC1 activation, which is important in muscle regeneration [210,211].

## 6. Concluding Remarks

The central dogma of biology changed with the discovery of functional ncRNA molecules, such as rRNAs and tRNAs. After many observations were initially treated with distrust by the scientific community, the importance of these ncRNAs was demonstrated by their functionality, mainly in gene expression regulation. 

The study of ncRNAs involved in gene expression regulation started with the discovery of small RNAs capable of binding to the 3’ UTR region of mRNAs, thus controlling protein production. The well-known miRNAs are associated with physiological processes, chronic pathologies, and infectious diseases. The emergence of long RNAs without an apparent ORF soon confirmed the presence of lncRNAs, involved in some of the same processes as miRNAs. In this context, many studies rapidly evaluated the regulatory functions shared by these molecules in signaling or metabolic pathways. Many studies demonstrated the interaction and regulation between these molecules and provided a new perspective on the ncRNA world. lncRNAs revealed their complexity when they were described as able to interact simultaneously with diverse macromolecules, controlling different points of gene expression flux. We encourage the study of different subsets of ncRNA in different models given the importance of their coregulation.

However, the annotation of these lncRNAs as non-coding is controversial. New studies have demonstrated that some can be translated into micropeptides that exert biological functions. The complexity of these molecules involves the capacity to interact with many types of molecules within the cell, and then the possibility of translation into peptides, increasing the regulatory capacity. Both forms are active and seem to be part of a regulatory network not yet completely described. We still need to understand how cellular components, or the molecule itself, are capable of controlling the timing of its translation.

In describing the variety of lncRNAs classes, the complexity exceeds the description of their chromosomal loci, used as the default in classification. lncRNAs can also be related to their ability to interact with different molecules, defining their involvement in the regulation of gene expression. The ability to acquire secondary structures mediates these interactions, such as the formation of circular structures, highlighting the multifunctional characteristics of these molecules. For much of the transcriptomic data deposited in databanks, no attempt has yet been made to analyze lncRNA regulation. Therefore, groups with interest in this area are encouraged to use pipelines developed to perform this analysis [212], for prospecting and further validating their functionality in different models.
